# Training a Continent: A Process Evaluation of Virtual Training on Infection Prevention and Control in Africa During COVID-19

**DOI:** 10.9745/GHSP-D-22-00051

**Published:** 2023-04-28

**Authors:** Suzan Joseph Kessy, Giorgia Gon, Yewande Alimi, Waheed Aryio Bakare, Katherine Gallagher, Emilio Hornsey, Lizzi Sithole, Ezinne Victoria Chinemerem Onwekwe, Tochi Okwor, Adekemi Sekoni, Alice Vahanian, Anna Vorndran, Thaddee Niyoyitungira, Tajudeen Raji, Chikwe Ihekweazu, Mohammed Abdulaziz, Folasade Ogunsola

**Affiliations:** a Infection Control Africa Network, Cape Town, South Africa.; b London School of Hygiene & Tropical Medicine, London, United Kingdom.; c Africa Centres for Disease Control, Addis Ababa, Ethiopia.; d Public Health England, London, United Kingdom.; e Nigeria Centre for Disease Control, Abuja, Nigeria.; f University of Lagos, Lagos, Nigeria.; g Independent consultant, London, United Kingdom.

## Abstract

**Background:**

Strengthening infection prevention and control (IPC) capacity was identified as a key intervention to prepare African Union member states to curb the COVID-19 pandemic. As part of the Africa Taskforce for Coronavirus, which helped implement the Africa Joint Continental Strategy for COVID-19 Outbreak response, the IPC Technical Working Group (IPC TWG) was convened to coordinate the development of IPC core components for preparedness, response, and recovery from COVID-19. As part of the IPC TWG’s work, the Africa Centres for Disease Control and Prevention, in collaboration with the Infection Control Africa Network, delivered virtual IPC training sessions targeted to African Union member states. We aimed to undertake a process evaluation of this training to inform and improve both ongoing and future programming.

**Methods:**

The scope of the evaluation was agreed upon through discussion with the training organizers and advisory members and a design workshop. A mixed-methods approach was used; data collection was partly prospective and partly retrospective due to the rapid start of some of the training activities. Existing available data included: usage analytics, the content of questions posed during the webinar and community of practice, and participant feedback survey results. In addition, in-depth qualitative interviews were conducted with a sample of webinar participants.

**Results:**

The rapid development of this training was efficient and responsive. The training reached more than 3,000 participants across the 2 rounds, but the numbers varied substantially by location. Participants engaged well during the question period during each webinar, but the asynchronous community of practice was less utilized during the evaluation time frame. Many participants appreciated the African focus of the webinars and gave positive feedback on the practical and context-specific content.

**Conclusions:**

The move toward online training provides an important opportunity to improve IPC across the African continent.

## Abstract (precis)

Virtual training programs provide a feasible way to strengthen infection prevention and control in Africa.

## Teaser

**Key Findings:**

- Participants specifically appreciated the context-specific adaptations and the African focus of the training.
- Using different modes of learning (e.g., offline materials, live webinars, and question-and-answer sessions) was essential for successful delivery of the virtual training.
- Results show that large-scale training on infection prevention is feasible at the fast pace required during the pandemic but requires proactive monitoring and ongoing adaptations.

**Key Implications:**

- Large-scale training on infection prevention is feasible at the fast pace required during the pandemic, but it requires proactive monitoring and ongoing adaptations.
- The feasibility of virtual training should be exploited in the future to support in-person training and enhance emergency preparedness on the African continent.
- Collaborative relations between multiple institutions strengthened the development, dissemination, and evaluation of this training and should be optimized in any future large-scale emergencies.

## BACKGROUND

Infection prevention and control (IPC) in the health care settings is a fundamental part of COVID-19 patient management to prevent infection from spreading to health care workers and health care users. Across low- and middle-income countries, prevalence of health care–acquired infections is 15.5%.^1^ Health settings have the potential to amplify outbreaks, as occurred in the United Kingdom during the early phase of the COVID-19 response.^2^ Key IPC measures recommended by the World Health Organization (WHO) include hand hygiene, respiratory hygiene, environmental hygiene, patient isolation, and use of personal protective equipment.

In many African countries, IPC practices are inadequate, including poor compliance with basic practices and inadequate systems and infrastructure for IPC.^3^^–^^5^ A critical need for training was identified from recent global surveys and IPC expert consultations.^6^^–^^8^ In 2020, the Africa Centres for Disease Control and Prevention (Africa CDC) established the Africa Taskforce for Coronavirus (AFTCOR) to coordinate the Africa Joint Continental Strategy for COVID-19 Outbreak pandemic response. The IPC Technical Working Group (IPC TWG), an integral part of the AFTCOR, was tasked with strengthening IPC capacity as a key priority intervention to prepare and equip African Union member states to curb the COVID-19 pandemic. It was also acknowledged that COVID-19 presented an opportunity to strengthen IPC in the African continent.^9^ To achieve this goal, the Africa Centres for Disease Control and Prevention (Africa CDC), in collaboration with the Infection Control Africa Network (ICAN), has been delivering IPC training programs across the African Union member states.

In the context of a global lockdown, these existing IPC training programs needed to be reviewed and adapted for online delivery.^10^^,^^11^ Other agencies, including the WHO, created an open learning platform on COVID-19 topics that demonstrated the core benefits of massive web-based learning, such as equity of access, flexibility based on self-paced learning, and quality of content that could be regularly updated based on the most recent evidence.^12^ Project HOPE developed a training-of-trainers model to enhance knowledge of COVID-19 concepts that was delivered to 4,291 personnel across 55 countries in 2021.^13^ Other examples include more targeted efforts, such as a pilot of mobile-based training for health workers in Nigeria that has effectively used a learner-centered approach to improve IPC knowledge scores.^5^ An open learning platform on COVID-19 transmission was developed by the London School of Hygiene & Tropical Medicine (LSHTM), and their experience points to the strength of peer learning and participant discussion fora.^14^ When the AFTCOR training was being developed, these virtual education initiatives were in their early stages. Hence, there was a hesitancy on whether such an approach would work in the African context, especially regarding access to information technology devices and Internet connection. Yet, there was some evidence to suggest that at least mobile access was widespread in low-resource settings before the pandemic.^6^

### Virtual Training for IPC

The training organized by the IPC TWG consisted primarily of 2 main components: a series of webinars (delivered live through Zoom and recorded and posted on YouTube with materials shared via email) and a virtual community of practice (a discussion forum using the messaging app Telegram, which allows larger groups than the more frequently used WhatsApp). The training targeted anyone interested in the subject matter across the continent, from health workers to policymakers to field epidemiologists. Social media, country focal points, and existing email lists of IPC stakeholders in the region were used to advertise the training, which was open to anyone. The training intended to promote learning through webinars using prepared content and participant-led question-and-answer (Q&A) sessions after each webinar and the community of practice. IPC experts from Africa CDC, ICAN, UK-Public Health Rapid Support Team, U.S. Centers for Disease Control and Prevention, WHO Regional Office for Africa, WHO Regional Office for the Eastern Mediterranean, and WHO Headquarters provided practical answers to the questions asked. The rationale for the Q&As and community of practice is grounded in situated learning theory—which emphasizes learning in the context where skills are ultimately applied and hence aims to tackle context-specific challenges of health practitioners in their own facility—and it extended to community settings over the training development.^15^^,^^16^

We undertook a process evaluation of the virtual training provided by the AFTCOR IPC TWG to inform and improve both ongoing and future programming. Evaluating the extent to which an online delivery of this type of training “works,” notably with a potentially large audience, can generate lessons learned from the current pandemic for future ones.^12^^,^^14^^,^^17^^,^^18^

## METHODS

### Scope of the Evaluation and Evaluation Team

The evaluation was a joint venture between the training organizers, which included members of ICAN, Africa CDC, and the UK–Health Security Agency, as well as LSHTM researchers. The scope of the evaluation was agreed upon through discussion with the training organizers, and an evaluation design workshop was held involving the members of the LSHTM Centre for Evaluation. The evaluation focused on the first 2 rounds of the virtual training delivered between April and July 2020 because this is the crucial moment—at the start of the pandemic—where key lessons from the training (content and modality) had to be drawn.

We used the UK Medical Research Council framework^19^ for process evaluation of complex interventions. Hence, our results are structured using the following themes: dose and adaptations, reach, mechanisms, and context. Research questions were synthesized under each theme presented in the results section.

1. How was the training format and content developed and adapted over time?
2. Were the training sessions delivered as intended?
3. How many participants did the training reach, and how does it compare across training rounds? Who has the training reached in terms of participants’ demographics and geography? Is information being shared beyond the direct recipients?
4. Do participants engage with the community of practice and Q&A? How does this vary over time?
5. Has the training enhanced participants' knowledge/skills? To what extent is the information new, confirmatory, or contradictory to other sources available?
6. Do participants feel able to translate their acquired knowledge/skills to their context? What would help them to do so? What barriers do participants face when implementing the recommendations of the training sessions in their contexts?

Additional focus areas of improvement arose directly from the internal feedback mechanism put in place by the training organizers further described later.

A mixed-methods approach was used; data collection was partially prospective and partially retrospective due to the rapid start of some of the training activities. The scope of the evaluation aimed to be responsive to the needs of the trainers while minimizing data collection to meet the organizers’ and participants’ limited capacity during the global pandemic.

### Data Sources

We used available data from: (1) usage analytics (e.g., online views of the webinar recordings and webinar participants’ demographics); (2) the content of questions posed during webinar Q&A sessions and the community of practice; and (3) the results of a feedback survey sent to participants after each webinar (Round I=592, Round II=354 responses across all 6 webinars of each round—this is the final number after data cleaning and removing duplicates).

In-depth qualitative interviews were conducted with a sample of webinar participants. All registered webinar participants based on the African continent with a self-reported IPC, patient safety, or quality officer role (the priority audience identified by organizers) were invited by email to participate in a short (30–40 minute) interview by ICAN/Africa CDC. Views on training content, access, usefulness, and sharing of training knowledge/materials with others were explored using a topic guide (Supplement 1). Interviews were conducted in French or English. In June 2020, 1,000 participants from the English webinar and 700 from the French webinar were invited to interview: 17 responded; 3 were excluded based on their specified job role; and 10 ultimately consented (7 in English, 3 in French). In August 2020, a further 180 English webinar participants and 45 French webinar participants were invited to interview: 13 responded and 8 consented to interview (5 in English, 3 in French). For this second round of interviews, we sent the invitation to a narrower pool to increase responses from regions with high participation but from which we had gathered few responses in the first round of interviews, specifically Southern Africa, Central Africa, and Eastern Africa. In total, 18 interviews were conducted across the 2 rounds.

A virtual focus group discussion was conducted with the training organizers using a topic guide (Supplement 2) to document the process of the training development and adaptations. The results presented here also rely on virtual unstructured observations of researchers during organizational meetings who were simultaneously involved in the training organization and evaluation.

### Analysis

We used descriptive statistics to summarize the usage analytics and the results of the internal feedback survey. Questions and answers posed during training sessions (webinar Q&A section) and in the community of practice were extracted and thematically coded in Excel. A combination of inductive and deductive methods was used to analyze qualitative data. Qualitative data from interviews and the focus group discussion were extracted into Excel and synthesized thematically. Themes were agreed upon before the extraction by the group conducting qualitative interviews and analysis. We do not have information on the countries of those who engaged with the Telegram group or the survey responses.

All available quantitative data supplied for the analysis were anonymized and de-identified. For the qualitative interviews, participants gave informed consent via email using the consent statement described in Supplement 3. Participants gave their verbal consent to have phone interviews recorded. Participants of the focus group discussion gave their verbal consent to participate and record the discussion. Recordings were made with a General Data Protection Regulation compliant software and were destroyed after data extraction. Transcripts were anonymized with unique identification numbers. No names or locations, apart from country, were mentioned in transcripts. Qualitative data were not shared, as this could have led to a breach of confidentiality.

### Ethical Approval

Ethical approval for this project was received from the LSHTM ethics committee, and Africa CDC provided a letter of support.

## RESULTS

### Dose and Adaptations

#### How Was the Training Format and Content Developed and Adapted Over Time?

The training organizers reported that the virtual training aimed to build capacity in IPC on the African continent to respond to the COVID-19 pandemic. They wanted the training to be practical, allow for context specificity (considering that different countries were at different stages of their response to COVID-19), and to be “Africa centric.”

*The aim was to teach people about basic principles so they can contextualize in their own environment- things are not always available; so, what is the next best alternative? Ensuring they can adapt/innovate locally. We also wanted to share what others have done.* —Training organizer

The IPC training was intended to be practical, adaptable for different country contexts, and be Africa centric.

The initial target audience included health workers who provide care directly to patients; IPC focal points; workers with water, sanitation, and hygiene responsibilities in health facilities; and Africa CDC rapid responders and volunteers based in country. Efforts to reach the target audiences were made through email networks of the organizations involved (Africa CDC and ICAN) and informal channels available to the organizers, such as professional WhatsApp groups, national IPC focal points, and social media engagement. From the Round II feedback survey, we have information on how respondents found information on these webinars. Of the 246 responses to this question (75% of the 344 responses to the survey for Round II, both languages; data not available for Round I), one-third found information about the training via email, 26% were recommended by a friend/colleague, 21% found information on the Africa CDC website, 14% through social media, and 3% through other means.

There was no formal enrollment or assessment of eligibility, and delivery was adapted to target a wider audience **(**e.g., managers and policymakers) between Round I and II in recognition of the heterogeneity of attendees.

*Given the virtual component of the training – we realized the ability of the webinar to be wider…And translated to something for all levels of the health system. We also tried not to lose those with no background in IPC. … [We] focused more on those (anybody) who gives advice.* —Training organizer

**FIGURE 1.**
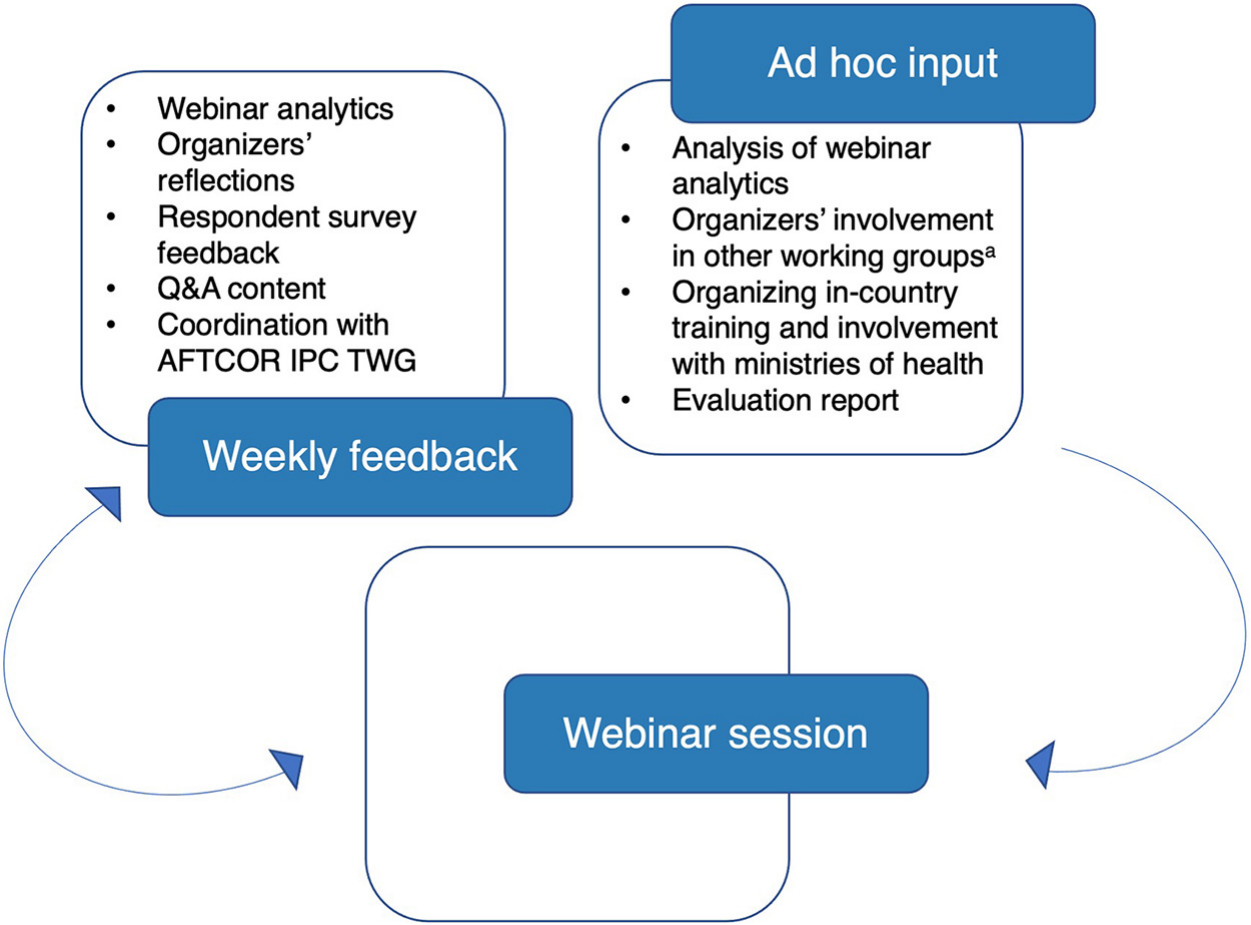
Sources of Feedback That Influenced Training Content in Real-Time Abbreviations: AFENET, Africa Field Epidemiology Network; AFTCOR, Africa Task Force for Coronavirus; IPC TWG, Infection Prevention and Control Technical Working Group; LSHTM, London School of Hygiene & Tropical Medicine; PHE, Public Health England; U.S. CDC, United States Centers for Disease Control and Prevention; WHO, World Health Organization. ^a^ Organizers’ involvement in other working groups and advisory channels included in particular: International agencies: WHO Headquarters, WHO Regional Office for Africa, WHO Regional Office for the Eastern Mediterranean, AFENET; Country agencies: U.S. CDC, PHE; Research groups: LSHTM.

**FIGURE 2:**
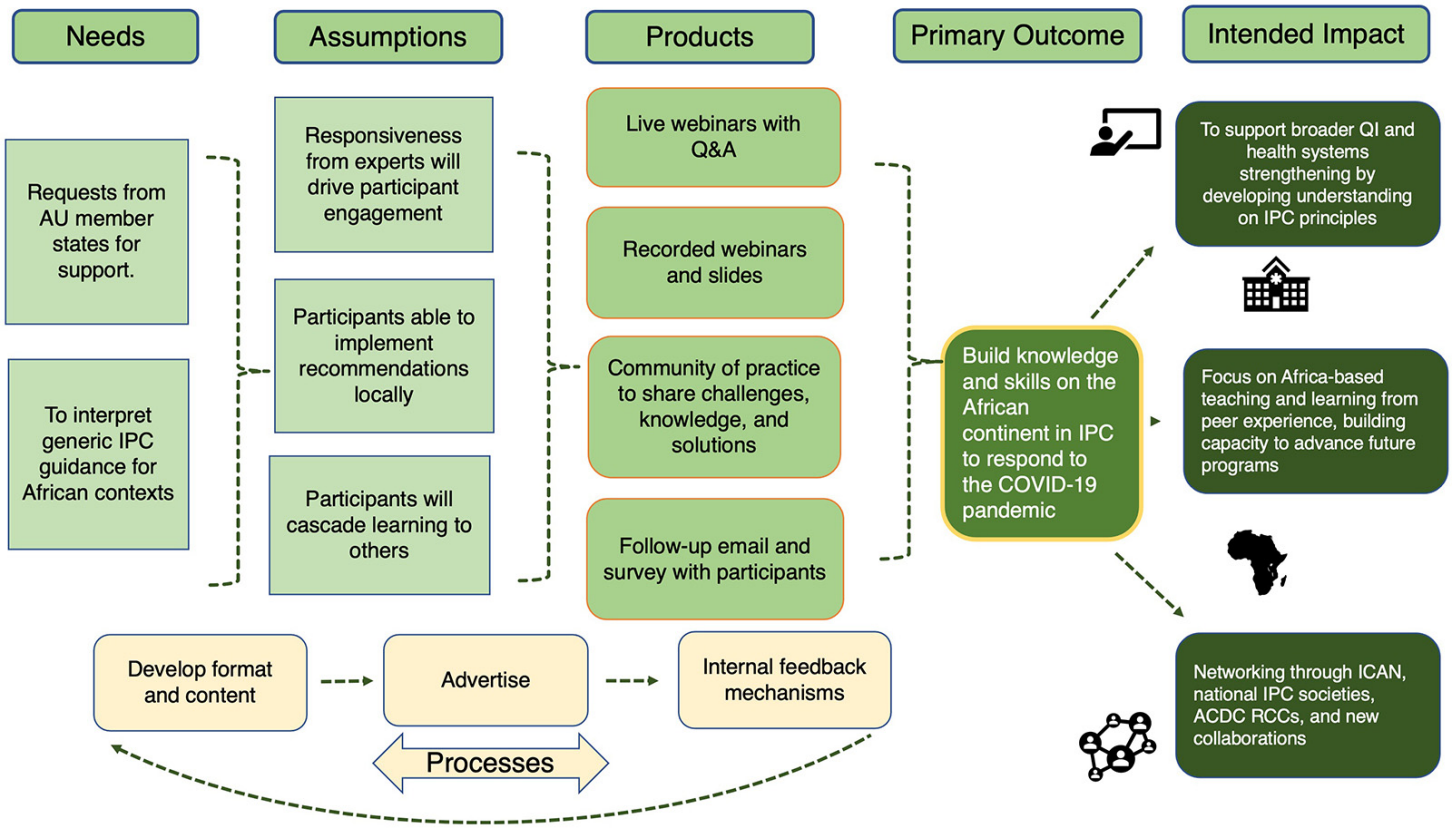
Simplified Theory of Change From Training to Intended Impact Abbreviations: AU, African Union; ACDC RCC, Africa Centres for Disease Control and Prevention Regional Collaborating Centre; ICAN, Infection Control Africa Network; IPC, infection prevention and control; QI, quality improvement.

The content was based on the ICAN training curriculum with adaptations to emphasize key priorities for COVID-19 response (Table 1; material can be found online: https://uk-phrst.tghn.org/resources/ipc-training-africa-cdc-en/). Before the travel restrictions and national lockdowns, the AFTCOR IPC TWG had organized 2 in-person training-of-trainer sessions in Nigeria and Côte d’Ivoire with representatives of 36 African countries as part of the COVID-19 preparedness activities. This training material informed the content of the virtual webinars. A system of internal feedback (Figure 1) enabled organizers to constantly evaluate and reassess the scope and reach of the webinars and was seen as a key part of the content development by the organizers. The internal feedback process led to organizers identifying 5 focus areas of improvement to increase the reach and quality of the training (Table 2). Overall, from the participant feedback survey, respondents found the clarity of information high, the content useful, and the quality of interaction good. The median of all scores was 4 and above out of 5, with no obvious differences between sessions, rounds, and webinar language (Supplement 4). This resonated in all qualitative interviews. The final theory of change reflects the activities and intended outcomes by the end of the second round of webinars (Figure 2).

**TABLE 1.** List of Webinar Dates and Topics^a^

**Round I – Date**	**Topic**
April 16, 2020	IPC Introduction: Overview and Preparedness
April 22, 2020	Triage and Patient Flow
April 29, 2020	Personal Protective Equipment for COVID-19
May 6, 2020	Isolation and Cohorting
May 13, 2020	Environmental Hygiene
May 20, 2020	Infection Control Measures in the Community
**Round II – Date**	**Topic**
June 3, 2020	Assessing IPC Preparedness
June 10, 2020	Environmental and Equipment Hygiene
June 17, 2020	IPC in the Community
June 24, 2020	Personal Protective Equipment for COVID-19
July 8, 2020	Handling Dead Bodies and Burials During COVID-19
July 15, 2020	COVID-19 and Transportation Industry

Abbreviations: Africa CDC, Africa Centres for Disease Control and Prevention; IPC, infection prevention and control.

**^a^**COVID-19 data source is the Africa CDC Outbreak Briefs (https://africacdc.org/resources/?wpv-resource-type=outbreak-briefs&wpv_aux_current_post_id=217&wpv_view_count=549).

**TABLE 2.** Examples of How Internal Feedback Led to Adaptation of the Training Aims, Format, Audience, and Content

**Identified Issue From Feedback**	**Identified Focus Area of Improvement**	**Action Taken**
Webinar analytics showed poor attendance from Francophone countries	To increase participation from Francophone countries	Changed the time of French webinars to cater for a more preferred time. Ensured translation of any prepared presentation materials
Questions raised during panel discussions consistently drew on informal/unsystematic knowledge of organizers	To increase space to discuss context-specific adaptations	Changed the webinar format to include a specific section each week where a different country would share their experience on the topic discussed to provide a real-world example and stimulate structured discussion
Webinar analytics showed relative poor attendance from Central and East Africa (predominantly West African attendees)	To increase participation beyond West Africa	Conducted additional promotion through existing channels
Weekly meetings among organizers and the internal evaluation report between Round I and II highlighted high traffic of irrelevant discussion on the COP	To improve engagement with the COP	Implemented a rota among the organizers to moderate the COP daily with regular posting of relevant information or documents to stimulate relevant discussion
Webinar analytics identified relatively few in a manager role and this was raised during Q&A in panel discussions	To increase participation of “managers and policymakers”	Edited content and prepared a specific “IPC for managers” webinar series currently being released

Abbreviations: COP, community of practice; IPC, infection prevention and control.

#### Were the Training Sessions Delivered as Intended?

Overall, the organizers felt that the training was delivered as intended. When the training was initially conceived in a concept note, the intention was to provide a 6-week “course” that would be repeated 4 times with different cohorts. Feedback during the initial 6-week course and evolving evidence led to an adaption of this. Some sessions were repeated where new guidance was being produced, and new sessions were added based on identified needs. Each 6-week cohort became an administrative block for the course implementation team rather than an integrated course. The webinars were intended to both integrate participants’ knowledge based on a single topic, as well as be more comprehensive for those attending multiple ones. The nature was intended to be flexible to accommodate various needs of participants across the continent based on their own needs.

The organizers reported that time constraints were the main barrier to ideal implementation. Organizers had several roles in the COVID-19 response, and there was little time for planning and securing webinar facilitators. Financial constraints meant that simultaneous translations in other key languages on the continent, such as Arabic and Portuguese, were not feasible. The French webinar was organized separately as it was less expensive than securing simultaneous translation of the English version.

The organizers reported that time constraints were the main barrier to ideal implementation.

### Reach

#### How Many Participants Did the Training Reach, and How Does it Compare Across Training Rounds?

Specific questions for focus areas of improvement included: Did the number of French viewers increase from Round I to Round II? Has the audience changed from Round I to Round II? Did participation from East and Central Africa increase from Round I to Round II?

**FIGURE 3:**
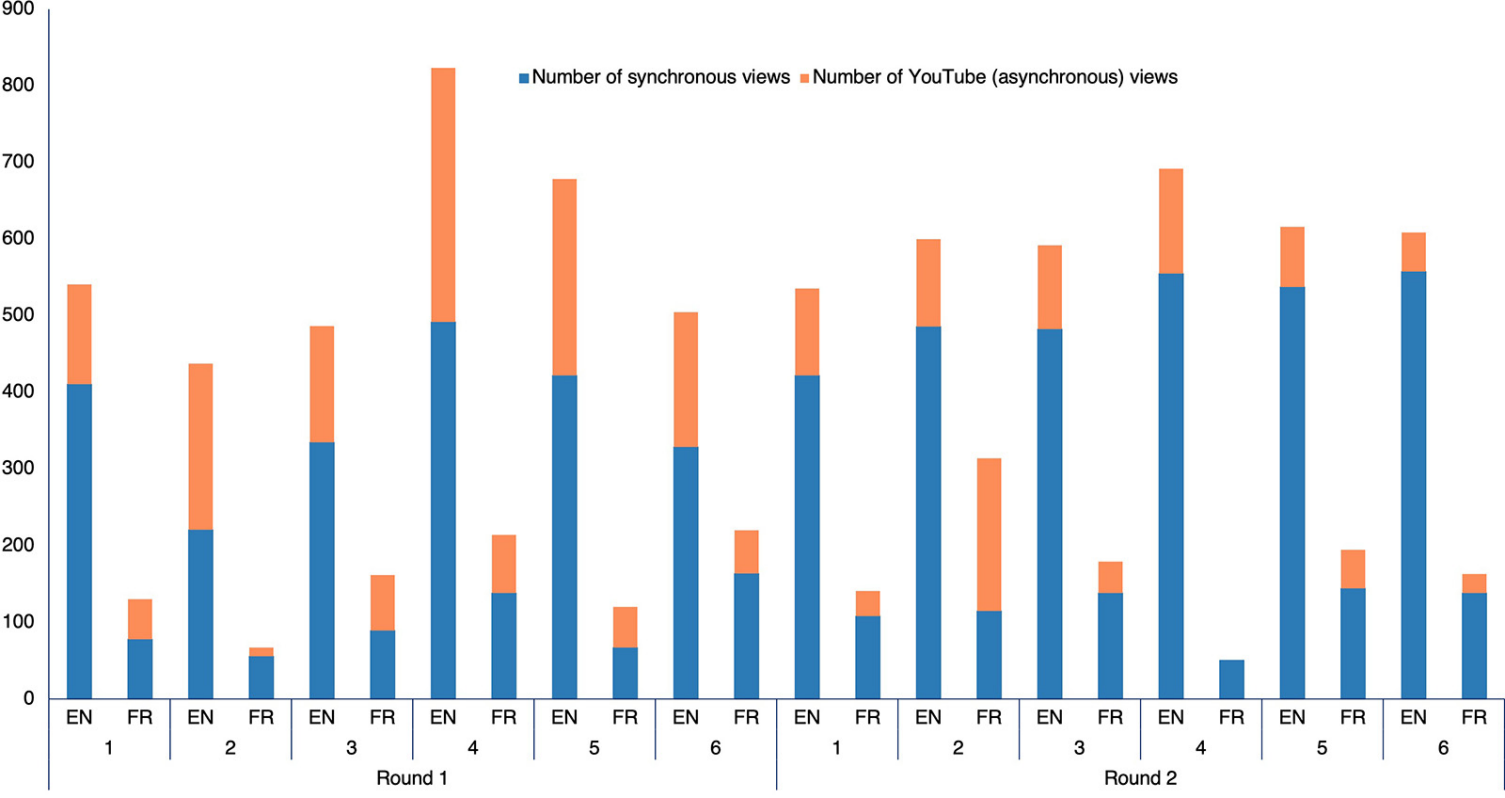
Number of Viewers and YouTube Views, by Language and Session

There were 1,751 individual participants in Round I and 1,672 in Round II who attended at least 1 webinar. In both rounds, less than 2% of participants attended all 6 sessions. Regarding the number of live views or viewers (the same participants can be counted multiple times if they participated in multiple webinars), Round I had 2,808 viewers, of which 21% attended the French version, and Round II had 3,657 viewers, of which 17% attended the French version. The number of viewers increased weekly for both the English and French webinars (Figure 3). The median time spent by viewers on the webinar increased from Round I to Round II for both languages. French webinars increased from 41 minutes (interquartile range [IQR]=6–74) to 56 minutes (IQR=25–86); English webinars increased from 43 minutes (IQR=10–74) to 48 minutes (IQR=17–78).

Additionally, 2,980 YouTube views of recorded sessions were logged as of April 22, 2021; 62% of these were from Round I. There was a high volume of clicks (2,366) on the online webinar slides in Round II (no such data are currently available for Round I), but there is no clear pattern over the weekly sessions (data not shown). We could not evaluate how the same participants engaged with different training components as we could not cross-reference attendees across different platforms.

Among respondents to this question in the participant feedback survey, 236 of 344 (68%) in Round II stated they had shared the webinar information with approximately 4,200 other people in their networks, suggesting information was widely shared by this self-selected group.

**FIGURE 4:**
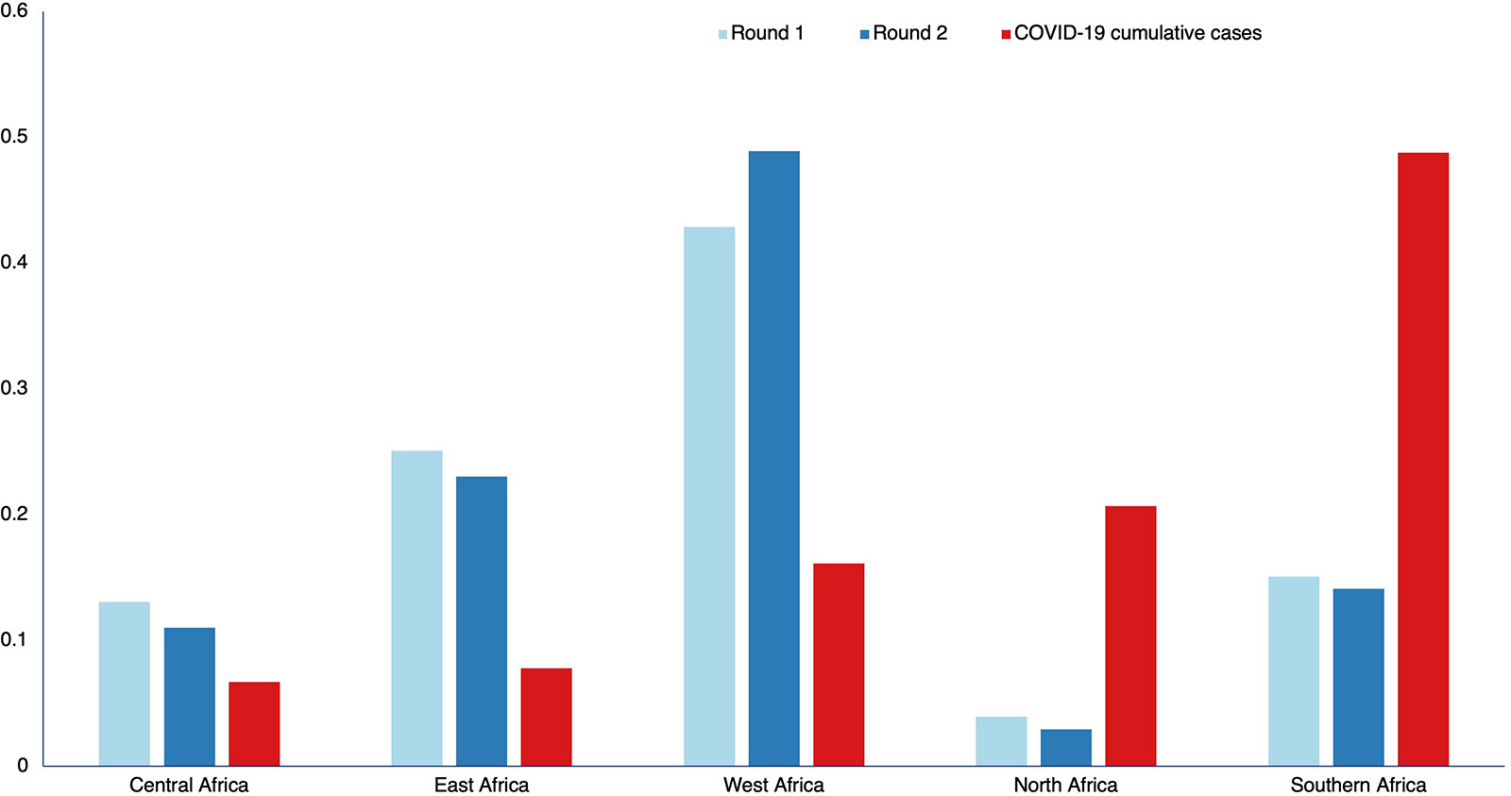
Proportion of Webinar Viewers and COVID-19 Cumulative Cases, by African Region^a^ **^a^**COVID-19 data source is the Africa CDC Outbreak Briefs: https://africacdc.org/resources/?wpv-resource-type=outbreak-briefs&wpv_aux_current_post_id=217&wpv_view_count=549

Representatives from 91 countries participated across the 2 rounds from all world regions. Those participants based on the African continent were the overwhelming majority: 87% in Round I and 88% in Round II. The geographical distribution across the African continent was similar in the 2 rounds (Figure 4). Participants from 51 African countries attended at least 1 webinar in Round I or II, translating to at least 1 participant from over 90% of the countries on the continent. West African countries had the highest number of participants in both rounds. Participation from Eastern and Central Africa did not substantially change between rounds. North Africa was the least-represented region in spite of having the second-highest number of COVID-19 cases during Rounds I and II.

**FIGURE 5:**
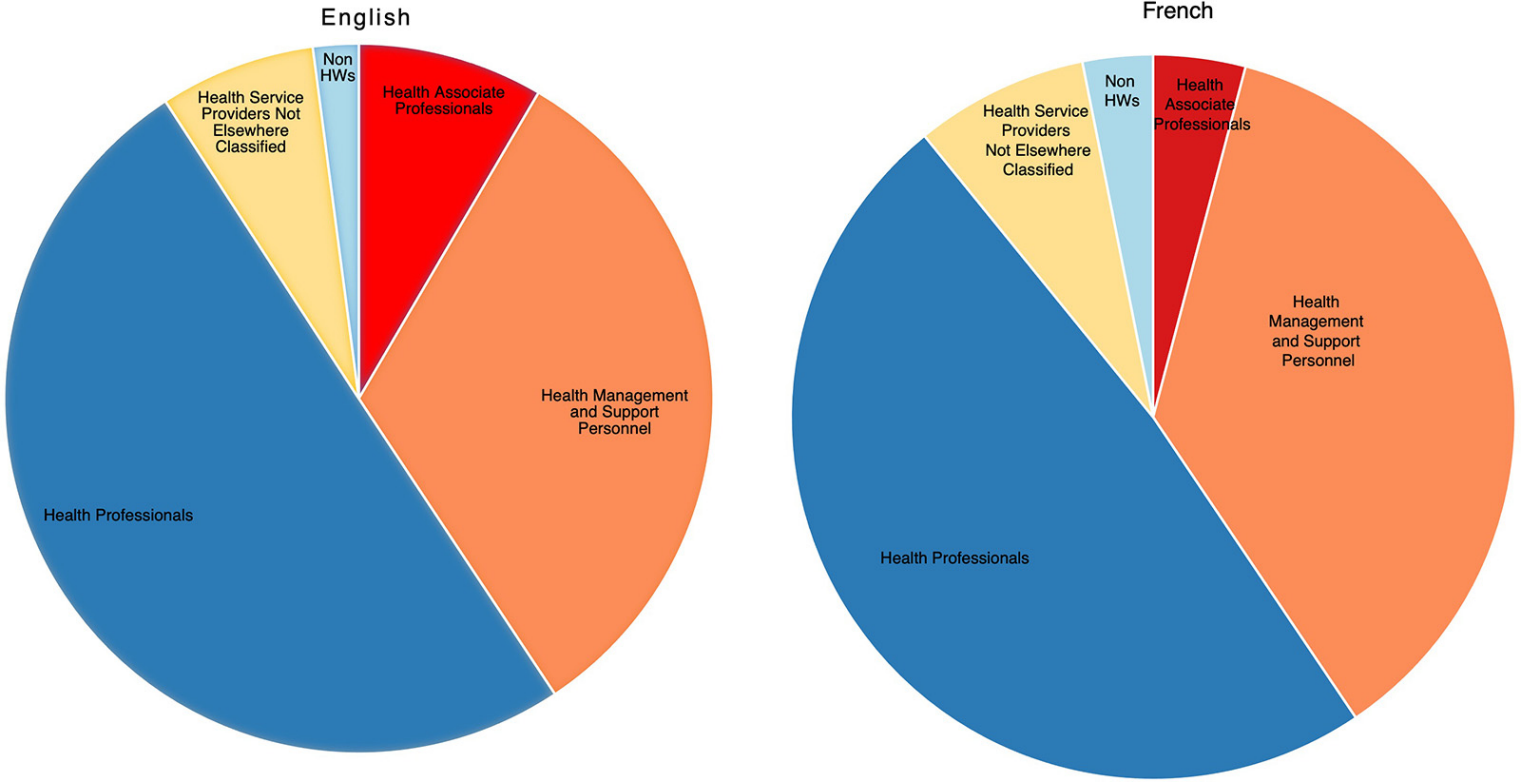
Individuals Who Registered for Webinars, by Profession Abbreviation: HW, health worker. ^a^ N=2,068 participants from Rounds I and II in the English webinars; N=1,021 participants from Rounds I and II in the French webinars.

We categorized the profession of participants (1,751 individual participants in Round I and 1,672 in Round II) based on a WHO classification for the health service.^20^ The distribution does not vary substantially for the French and English participants (Figure 5) or between Rounds I and II (data not shown). Overall, one-half of the participants were classified as health professionals (e.g., doctors and nurses); about one-third were classified as health management and support personnel (e.g., hospital managers and accountants); 4%–9% were classified as health associate professionals (e.g., laboratory staff); 7%–8% were classified as health service providers not elsewhere classified (e.g., volunteers); and about 2%–3% were classified as non-health workers, (e.g., architects and journalists).

In the qualitative interviews, most respondents stated they had preferred to watch the webinars live and valued being able to pose questions in real-time or take part in the live discussion; however, it was clear that the recordings were useful to confirm issues after the live webinar was over (e.g., if they had problems understanding the language or if the volume of the information had been high). Approximately one-half of the interviewees stated they had downloaded the materials sent after the webinar (documents and presentations). All interviewees had shared some elements of the training with colleagues (PowerPoint slides, documents, and/or YouTube links to webinar recordings). Four interviewees mentioned they had used the information in the webinars to conduct formal training sessions with large numbers of health care professionals as part of their job role; one respondent reported regularly sharing important aspects of the training sessions at hospital management meetings.

*I can tell you in my facility, [among] our health care workers we’ve only had 3 people that have actually been infected because of some of the information that I have gathered from the webinar, which I go back and then apply.* —Key informant

*Every week I train people, I have shared what I learned from Africa CDC and WHO. I trained 33 people last week, tomorrow I will travel and train people for face-to-face training.* —Key informant

#### Do Participants Engage With the Community of Practice and Q&A?

Most respondents stated they had preferred to watch the webinars live and valued being able to pose questions in real-time or take part in the live discussion.

Specific focus areas of improvement included how did engagement vary over time and has the participation in the community of practice and Q&A increased from Round I to Round II?

**FIGURE 6:**
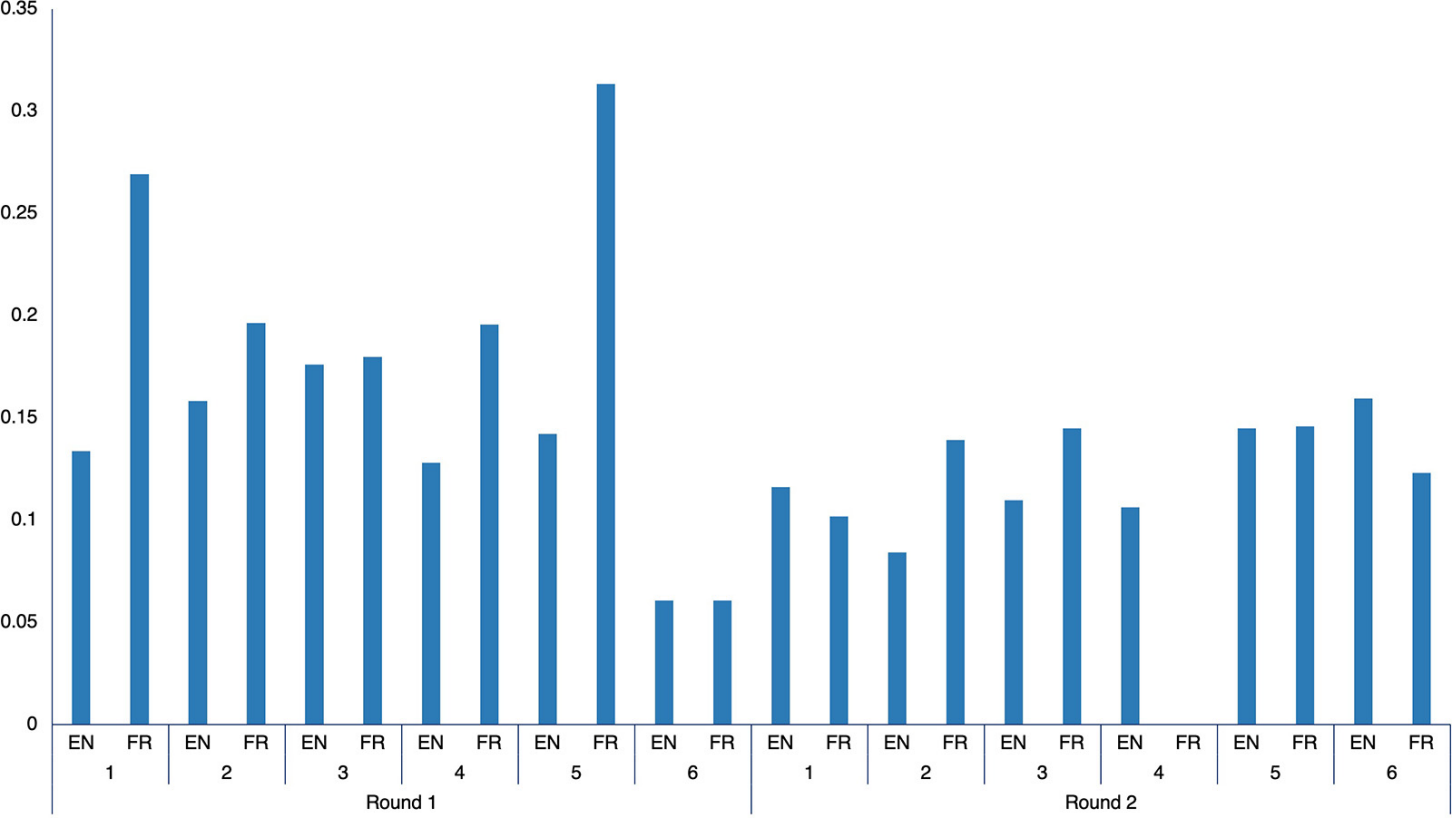
Proportion Of Webinar Participants Who Posed Questions During Live Q&A^a^ ^a^ Empty column for French session 4 in Round 2 indicates that the data were not available for that session as run on a different software compared to other sessions.

Among the attendees, 13% (842 of 6,465) posed questions during the webinars. The second round of webinars included a presentation section that involved a direct country perspective after the main topic; this allowed less time for questions and may explain why participation was lower in Round II (Figure 6). Participants were directed to use the community of practice to ask questions after the session or to see replies to questions that could not be addressed live. A summary of the questions and relevant answers was compiled and posted online (https://africacdc.org/download/covid-19-infection-prevention-and-control-your-questions-answered/).

There were 2 separate communities of practice established through Telegram: 1 in English and 1 in French. Between April 28 and August 1, 2020, there were 686 users and a total of 3,326 messages on the English platform, of which 23 were technical questions/comments posed by participants. The remainder of the messages were social well-wishes (saying hello), gratitude to the session organizers, or queries over the logistics of accessing the webinar sessions. All but 5 “answerable questions” were answered by the organizers. The French community of practice steadily built up a membership of 208 participants and yielded a total of 1,248 messages, of which 68 were technical questions/comments (including sharing guidelines), and 39 were additional questions transferred from the Q&A section of the live webinars. All “answerable questions” were answered by the organizers with further discussion by participants. The remainder were social interactions or discussions on specific topics broader than IPC (e.g., application of the One Health approach). Overall, the French Telegram was utilized more by participants compared to the English one. The proportion of the traffic that was relevant/technical remained low on both communities of practice but slightly increased from Round I to Round II (data not shown).

From the qualitative interviews, most interviewees had registered and used Telegram; 4 did not use it, of which 1 interviewee stated they didn’t know how to access it. Among those who had used Telegram, 3 stated it was useful, while others were more ambivalent. Overall, participants felt that there was some utility to the forum, but it included a very high volume of messages that were not content focused.

*l look for the links the messages I need. If you don’t open Telegram for 2 day you see 600 messages and some are saying hi and are here to socialize and others have technical problems with the app.* —Key informant

**FIGURE 7:**
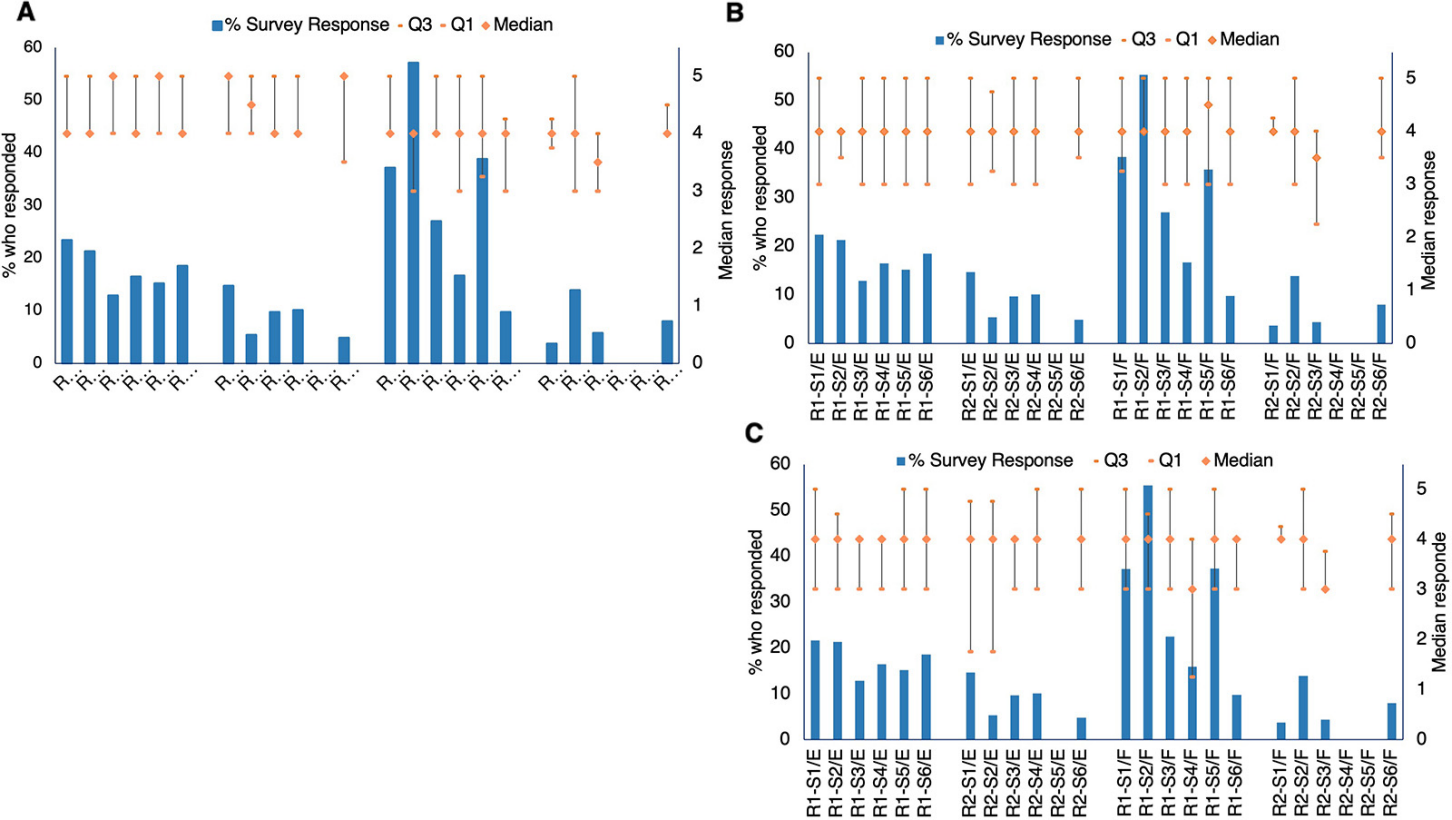
Proportion of Webinar Attendees Who Responded to the Question and Median of Responses to the Statements: (A) Instructions about subsequent training programs were communicated clearly; (B) The community of practice created on Telegram (COP) was a useful follow-up platform for me; and (C) Questions on Telegram (COP) were answered^a,b,c^ Abbreviation: COP, community of practice. ^a^Responses were out of 5; 5 indicated strongly agree and 1 indicated strongly disagree. ^b^R=Round; S=Session; E=English; F=French; Q=Quartile. ^c^Empty columns indicate that the survey data were not available for that session.

The internal feedback survey (Figure 7) suggested relatively high satisfaction with the community of practice (median between 4 and 5—except for R2–S3 for French, which scores between 3 and 4), but the proportion of respondents to these questions was low.

### Mechanisms

#### Has the Training Enhanced Participants' Knowledge/Skills?

A focus area of improvement included the inclusion of more context-specific experience sharing being valued by participants. About two-thirds of interviewees stated that the webinars had provided them with new information as well as confirming or “revising” knowledge they had acquired from other sources. Specifically, participants mentioned the following topics as new information with a COVID-19 focus: personal protective equipment application, environmental cleaning, case definitions, triage, and community strategies such as mask-wearing and social-distancing. One-third of interviewees stated the information received in the webinars had been simply confirmatory of knowledge acquired elsewhere, but the confirmation was reassuring in the face of the pandemic. All interviewees stated that the content aligned with the information they had received from other sources rather than contradicting it. However, a few interviewees recognized the nature of the emerging pandemic and how information evolved through the sessions, with some recommendations being less clear at the beginning and recommendations changing as the world learned more about COVID-19.

*For example, on proper use of PPE [personal protective equipment], based on a risk assessment. I understood this when I started to attend the webinar. But I didn’t know what do you mean by risk assessment. When I listen to one of the presenters she really explained it … in a table form. I have the slide with me.* —Key informant

All interviewees stated that the “Africa focus” to the webinars was useful due to common issues across the continent limiting their ability to respond to COVID-19 (e.g., limited resources and difficult supply chains). Interviewees mentioned that they valued the opportunity for peer-to-peer learning in the face of these limitations. However, several interviewees added a caveat to their response, mentioning that there were still some regional and country-specific issues that were not addressed with a continent-wide approach (e.g., specific cultural practices).

*A lot of new info through the experience of the other countries - so mainly the sharing of experience [was valuable].* —Key informant

Interviewees stated that the “Africa focus” to the webinars was useful due to common issues across the continent limiting their ability to respond to COVID-19.

Several participants mentioned how this is an opportunity to include IPC within in-service training across the continent.

*If IPC is properly incorporated into the system and if CDC Africa can push so that most medical schools have IPC as a compulsory module which every physician must validate to graduate, we will go somewhere.* —Key informant

**FIGURE 8:**
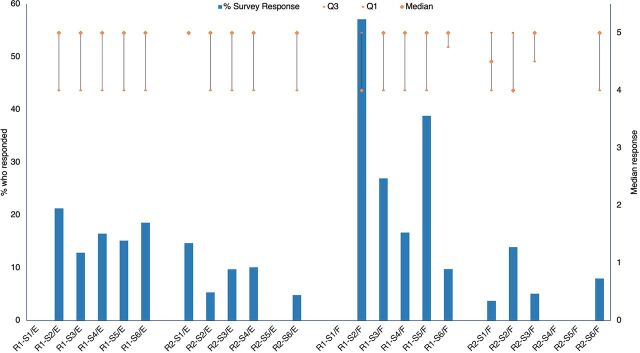
Proportion of Webinar Attendees Who Responded to the Question and Median of Responses to the Statement: “My IPC knowledge has increased”^a,b,c^ Abbreviation: IPC, infection prevention and control. ^a^Responses were out of 5; 5 indicated strongly agree and 1 indicated strongly disagree. ^b^R=Round; S=Session; E=English; F=French; Q=Quartile. ^c^Empty columns indicate that the survey data were not available for that session.

From the internal feedback survey, most respondents strongly agreed with the statement, “My IPC knowledge has increased,” (Figure 8) across all sessions and both French and English webinars, with no obvious differences by language, week to report, or proportion who responded.

### Feasibility Within Local Context

#### Do Participants Feel Able to Translate Their Acquired Knowledge/Skills to Their Context?

The range of questions raised during Q&As and in the communities of practice shows that many of the questions addressed context-specific challenges (e.g., How can health care workers protect themselves when there is little or no personal protective equipment available? How do we address staffing problems in settings with high HIV infection rates where health care workers are affected and fall within the vulnerable group that should not work in COVID-19 isolation facilities? Can a toilet be shared in a quarantine facility?).

All interviewees felt they had been able to act on at least some elements of the training. Interviewees stated the training had directly affected their response to COVID-19 in several ways (e.g., improving environmental hygiene in their workplace, increasing cleaning frequency, cleaning “depth” and materials used; improving waste disposal and hand washing practices; initiating a system to triage patients in the hospital setting; case reporting; and changing the set-up for their hospital isolation facilities). Some challenges that prevented participants from enacting the training included lack of space to adequately social distance, shortages of protective equipment or disinfectant, interrupted water supplies, and financial resources. Two respondents stated that behavior change at their institute had been slow despite training.

*[The trainings] are very easy to put into practice, because the people you have brought to the webinar are the people who have really done it in practice, they are not some professors somewhere without experience, they have really done it so I can translate it into my own workplace.* —Key informant

**FIGURE 9:**
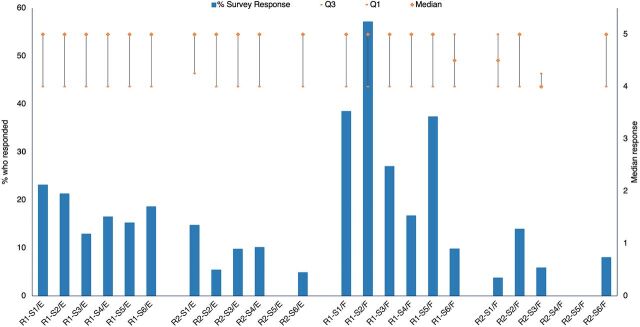
Proportion of Webinar Attendees Who Responded to the Survey and Median of Responses to the Statement: “I will likely improve/change my IPC practice as a result of the knowledge gained”^a,b,c^ Abbreviation: IPC, infection prevention and control. ^a^Responses were out of 5; 5 indicated strongly agree and 1 indicated strongly disagree. ^b^R=Round; S=Session; E=English; F=French; Q=Quartile. ^c^Empty columns indicate that the survey data were not available for that session.

Internal survey results suggested that respondents had a high level of confidence in the likelihood of improving/changing practice as a result of the training (Figure 9) across all sessions and across both French and English webinars with no obvious differences between languages or over time to report.

## DISCUSSION

The rapid development of this training, that included ongoing evaluation while the delivery occurred, was expedient and responsive to participants’ needs. It necessitated a flexible design with continued engagement from the delivery team and a good mechanism of internal feedback to respond to emerging needs in a rapidly evolving pandemic. The delivery was timely. Overall, we believe the underpinning evaluation was a strength of this training modality. The mixed methods approach we used allowed for data triangulation and was not just done for academic purposes but to improve the webinar series itself.

We believe a strength of this training modality was the underpinning evaluation, which helped to improve the webinar series.

The training reached more than 3,000 participants and more than 6,000 viewers across the 2 rounds and the various channels overall, with 87% being based on the African continent. The numbers varied substantially by location, with the highest level of participation in the West Africa region. The COVID-19 pandemic hastened digital access across Africa but also exacerbated inequalities among those who could not access good quality digital space, which is inevitably a key limitation of the training modality chosen here.^21^^,^^22^ In particular, the minimal uptake in Arabic-speaking countries—where there were a high proportion of COVID-19 cases during the training period—and in Portuguese-speaking countries highlights the need to include a wider range of Africa Union languages in similar initiatives in the future. Regarding engagement with situated learning opportunities, the Q&A sessions were very well utilized and praised by participants; in contrast, community of practice engagement was low in both rounds during the evaluation time frame and mostly used for nontechnical content. After Round II, the organizers used several strategies to successfully improve the community of practice engagement (Box); additional improvement could have included a regional (at country or subcountry level) community of practice. The value of the Q&A should be considered in future programming of similar initiatives. There was evidence of participants sharing the training content widely; this may have facilitated an amplification effect, but it is hard to quantify. The sharing of content was self-reported by a self-selected group (those who completed the survey or interview), and we do not know the depth or nature of any shared content.

BOX How Did Organizers Use This Evaluation to Improve the Virtual Infection Prevention and Control Training?

Africa Centres for Disease Control and Prevention (Africa CDC) has conducted additional rounds of virtual training since the rounds we evaluated in this article. We comment on developments in the program, particularly those areas that have seen substantial changes based on the results of this evaluation.

**Virtual Platforms**

Social media was exploited more comprehensively to improve accessibility on multiple platforms. As a result, live streaming of the webinar on Africa CDC social media handles (Facebook, YouTube, and Twitter) built momentum. The use of Facebook Live increased the webinar’s reach. In addition, it provided a more affordable means to join due to requiring lower bandwidth than Zoom and specific social media packages offered by Internet providers in some countries.

On both the English sessions on Wednesdays and French sessions on Fridays, the number of live views fluctuated between 200 and 2,000, with additional likes, shares, and comments. Participation on YouTube and Twitter remained lower than on Facebook, but the organizers continued to promote all platforms in the dissemination strategy. The questions posed in the comments section of live streams on social media platforms were monitored, but this placed a considerable burden on the hosts.

**New Simultaneous Translations**

Arabic and Portuguese interpreters were added to provide simultaneous translation to the English session for Round V. This provided opportunities for the speakers of those languages to participate, interact, and ask questions during the webinar. Since this was instituted, an increased number of participants from both Arabic- and Portuguese-speaking countries has been observed.

**Engagement With Community of Practice**

The English community of practice platform grew to more than 1,300 members, and the French version to more than 800 members at its height on March 3, 2023. The conversation became much more focused on infection prevention and control (IPC) issues as the registration and information technology issues were resolved. It had become more coordinated than when the evaluation was conducted, with daily input by moderators from Africa CDC. Members were encouraged to share only IPC-related issues, materials, and experiences, while experts on the platform provided solutions to the challenges shared. Individual cases were followed up through one-to-one support if required to avoid the platform being swamped with messages. Moderators posted a “question of the day” to spark discussion and drive interaction with members. Materials, including webinar presentations, guideline documents, videos, and charts, were shared to refresh memories, answer questions, or solve problems. Some members chatted directly with moderators or peers if they didn’t want to share information with the wider community of practice. All this effort resulted in a more dynamic and engaging community of practice platform, but it continued to require constant care and attention to maintain focus and utility for its members.

**Move to Focus on Managers**

From Round V onward, the training team decided to concentrate the webinars more on the specific concerns of managers (at all levels) in health systems because many health workers involved in IPC delivery already had the technical skills, but implementation remained a challenge without buy-in from managers. This focus also aligned with the need to integrate the COVID-19 response with longer-term systems strengthening and try to move away from IPC being regarded as a reactive measure for use only in outbreaks.

**Pivot to Broader IPC**

Overall, there have been 14 series of webinars delivered, and the program finished on December 15, 2022. The content was adapted over time to address other infectious diseases of concern, such as cholera, monkeypox, and Ebola virus disease, and to address more advanced IPC program delivery skills, including quality improvement, surveillance, and clinical audits. The total number of attendees (this is the sum of participants in each session; hence, some attendees may appear more than once in this sum) reached from May 2020 to December 2022 was 46,941.

Many participants appreciated the African focus of the webinars. Their appreciation increased as the content was practical and context-specific. This confirmed the value participants placed on the situated learning approach developed by the organizers. The focus on contextualized learning was a distinctive feature of the training, which distinguishes it from other mass open learning platforms available during the COVID-19 pandemic.^12^^,^^14^ Both the quality of the content and the degree of interaction between the content and the participants have been highlighted in predicting student satisfaction in the context of online learning.^23^^,^^24^ This was evident from all sources of data and, in particular, from both the interviews and the level and type of engagement with the Q&A. There is a tension between keeping a continental perspective throughout the webinars and including local contextual information. Regional training cannot respond to every specific circumstance, and AFTCOR continued to support country-level in-person training where possible, in addition to this virtual and remote delivery.

From the available data, it is very difficult to assess the degree to which participants’ level of knowledge or skills advanced or whether lessons learned were effectively deployed in the participants’ local context. Interviewees did express that there was a mix of confirming and building on previous knowledge, but we have no way of quantifying the depth or longevity of any learning. Self-reported improvements in knowledge were reported by survey respondents. For a few webinars, we attempted to assess knowledge more thoroughly with questionnaires before and after live webinars; however, the feasibility of this assessment method was limited and could not be pursued. More effort to systematically appraise knowledge gains would be a valuable addition to an evaluation of future programs; evidence from other studies^5^^,^^13^^,^^25^^–^^27^ suggests knowledge gain from virtual-based platforms is feasible, although gain may differ by health care group. The high numbers of offline viewers and clicks on the slides suggest that participants engaged highly with self-supported learning. This provides some, although weak, evidence that knowledge acquisition has progressed beyond the synchronous webinar activity. Interviewees also expressed a variable ability to influence their local context using the knowledge acquired; several contextual barriers were reported, including power, personnel, financial, and equipment barriers. While there was no expectation that education strategies alone would be successful in implementing behavior change,^27^ education is a necessary condition for motivated individuals, who, in the right enabling environment, can leverage behavior change.^28^

More effort to systematically appraise knowledge gains would be a valuable addition to an evaluation of future programs.

The COVID-19 pandemic stimulated a need for rapid and innovative ways to provide technical support and IPC trainings, especially in many low-resource settings where IPC practices are suboptimal. This was one of the first webinar series that targeted IPC practitioners in low-resource settings. Concurrently, attempts to reach a global audience on the topic of COVID-19 were ongoing^12^^–^^14^^,^^27^ and shared pedagogical similarities to the training described in our article, including a focus on the learner’s own pace and peer learning. The use of webinar-based courses is a quickly evolving educational approach, and many organizations have delivered these courses during the pandemic.^30^^–^^33^ Compared to another similar initiative aimed at training health workers in low-resource settings during the COVID-19 pandemic,^13^ we also found that this mode of delivery is less expensive and more time efficient than in-person training, especially in the context of rapidly evolving evidence.^12^ Offline educational material was particularly useful to health workers who faced competing priorities and technological challenges to joining live sessions.^12^^,^^27^ Retention, although not a direct aim of this program, was low across the sessions, as acknowledged in other virtual educational initiatives conducted during the COVID-19 pandemic.^14^^,^^34^

Various methods of encouraging live interaction and participation are possible (e.g., moderated questions^35^ or live social media interaction^30^); our intervention combined live webinars that included a strong Q&A component with building a community of practice. The team leveraged their combined technical and educational skills to deliver interactive webinars, but the community of practice required ongoing input to become a valuable resource for its members over time and, in particular, after the webinar rounds evaluated in this article (Box). Perhaps enabling people to connect socially across countries through sharing aspects of the self might not be at variance with a scientific program about professional practice at first.^36^ Curating and moderating such virtual groups requires skill and energy, but organizers felt it helped them to understand their audience and was a worthwhile addition to the program. As described for the broader idea of a community of practice, there are tensions between planning and spontaneity, action and principles, variance in how firmly members identify with the group, and the scale (local to global) in the making of any community of practice, and the organizers reinvented the community of practice weighing these tensions over time (Box).^37^ Overall, the flexible intervention design—integrating a combination of standard didactic teaching (live presentations/slides/recorded) and situated learning opportunities (community of practice and Q&A), including live and offline materials over a range of virtual channels and formats, and incorporating an integrated feedback mechanism—enabled the training team to meet the demands of as many participants as possible. The flexibility of design and delivery have been identified as key features of successful implementation in another training initiative, partly virtual, organized during the COVID-19 pandemic across 11 African countries.^27^

The webinar series aimed to be available to any individual who would benefit and to reach as many people as possible. Hence, it could not run through the standard trainer or training-of-trainers model or traditional cascade training, which Africa CDC typically conducted and continued to support in parallel. However, the training could have had a greater impact if national IPC programs and designated IPC focal points were stronger in country to conduct local training-of-trainers and provide in-person support, as tested successfully by 2 parallel initiatives including some of the same countries on the African continent.^13^^,^^27^ Overall, the lack of consistently strong country-based focal points in line with some of the structural gaps in low- and middle-income countries discussed earlier highlights the benefit of using an integrated approach to education and training.^6^ Tailoring the training more specifically to different professional cadres (nurses vs. doctors) would also have added value.^28^ Africa CDC was able to train some national IPC focal points before the COVID-19 pandemic, and this group of people became major IPC training facilitators during the lockdown when there were movement restrictions affecting international and national flights. Ensuring more such training is done outside of emergency situations can facilitate knowledge retention and the early cascading of training during emergencies.^38^ The world is experiencing an essential shift to remote learning that, with time, may evolve into a dominant method for practical reasons. Therefore, knowing when and how to promote which type of learning (in person vs. online or a combination) to maximize learning is a crucial component of emergency preparedness, and it has been argued that this is an unprecedented opportunity for medical education on the African continent.^27^^,^^39^

### Key Lessons and Recommendations

The training team exploited the momentum to strengthen IPC well beyond the COVID-19 pandemic. Key lessons learned and recommendations for future pandemics in the context of low-resource settings, which overlap or complement lessons learned from other mass online learning platforms,^12^^,^^13^^,^^27^^,^^34^ include the following.

- Develop strong collaborations with relevant agencies and partners such as ICAN, WHO Regional Office for Africa, WHO Regional Office for the Eastern Mediterranean, U.S. Centers for Disease Control and Prevention, and Africa Field Epidemiology Network.
- Adopt a blended learning approach: in particular, combine virtual training and in-person country-level support (training of trainers)—the latter was not evaluated in this article.
- Focus the training curricula on specific contexts with sharing of countries’ experiences.
- Continuously evaluate and review the training aim, content, format, and audience.

### Limitations

Limitations were largely due to the minimal preparation time available. We were not able to investigate the needs of the audience beforehand or to make the most of additional platforms such as social media. Unpredictable financial resources were also a limitation.

As far as possible during this evaluation, we worked with existing data to minimize any additional burden on participants or organizers. There were limitations in what was available to analyze. For example, we had no way of cross-referencing those who attended the webinars live and those who watched recordings on YouTube. Thus, it is difficult to estimate retention over the whole course when we do not know how many regular participants engaged over different platforms, which could give us information about the poor retention across sessions that we found. The emphasis during delivery was on improving reach, such as by using multiple platforms and minimizing registration information, but this has made evaluating some other outcomes more challenging. The low response rate from the internal survey and the interviews also limits the generalizability of our findings. We are aware that there is going to be a self-selection bias in those data sources toward those who were more positively engaged by the training. Unfortunately, we have no way of checking whether these respondents are similar regarding health care facility level or rural/urban status to the wider pool of viewers. Within this small, self-selected group of IPC practitioners, we reached data saturation during interviews. We strongly relied on data triangulation to provide robustness to our results, as recommended elsewhere.^29^

## CONCLUSION

As of December 2022, Africa CDC has conducted the 14th round of these webinars and has since tailored its focus to behavior change for IPC and IPC for other epidemics, such as Ebola virus disease, to match changes in the emerging IPC gaps on the African continent. We summarize key milestones in training development in the Box. Several interviewees in this evaluation remarked on the poor levels of IPC across the continent and the need for IPC to be higher on the agenda; the unprecedented reach of these online IPC training sessions on the continent has inaugurated this process.

## Supplementary Material

GHSP-D-22-00051-supplements.pdf

## Notes

**Peer Reviewed**

**First published online:** March 23, 2023.
