# Supplementary material for: Training a Continent: A Process Evaluation of Virtual Training on Infection Prevention and Control in Africa During COVID-19
Source: Glob Health Sci Pract. 2023 Apr 28;11(2):e2200051. doi: 10.9745/GHSP-D-22-00051 (PMC10141425; doi:10.9745/GHSP-D-22-00051)

Supplement to: Gon G, Kessy SJ, Alimi Y, et al. Training a continent: a process evaluation of virtual training on infection prevention and control in Africa during COVID-19. *Glob Health Sci Pract*. 2023;11(2):e2200051. https://doi.org/10.9745/GHSP-D-22-00051

Supplement 1. Interview guide

Preamble

Thank you for taking the time to talk to us today. My name is _____ and I'm helping Africa CDC and ICAN evaluate the effectiveness of the IPC webinar programme. None of the information we keep will be identifiable or traceable to you, the answers you give us will help us improve the webinar programme and help us understand more about how people are using the webinars and telegram community of practise. You wont get any payment for taking part in this interview and you're free to stop the interview at any time, we anticipate it will take about 30mins.

**Question**	**Why are we asking this**
What is your background/ job role?<br><br>Prompt: do you feel the content was relevant to your job/work at the moment?	Are they able to enact the learnings from the training
Where do you usually get information about IPC? Where have you obtained IPC information recently in the context of the COVID-19 pandemic?	Background knowledge/ other sources of info
Did the webinar provide new information or simply confirmation of other training you have done?<br>Did anything in the training contradict things you have read/ been taught in the past? If so where was this?	Dose and utility
How many IPC webinars did you attend and which ones?<br><br>Prompt: were these recommended? Or out of your own interest? Or because of practicalities?<br><br>Could you access materials after the webinar? How useful are these?<br>Did you access the live or youtube webinar? If so why, and do you engage with the Telegram/webinar?<br><br>Did you experience issues in accessing the webinar or Telegram, if so why? (prompt: lack of access to IT devices, bandwidth, time)<br>Do you think the TELEGRAM is a useful adjunct to the webinars? How could it be improved?	Utility

Supplement to: Gon G, Kessy SJ, Alimi Y, et al. Training a continent: a process evaluation of virtual training on infection prevention and control in Africa during COVID-19. *Glob Health Sci Pract*. 2023;11(2):e2200051. https://doi.org/10.9745/GHSP-D-22-00051

Are you able to enact what you have learned in the Webinars?<br>If so why ? or What things would help you enact the recommendations in the training?<br><br>Prompt; is your management supportive? / do you have professional support. Do you have enough physical resources/ can you mobilise resources?<br><br>Has attending the webinars affected the way you are responding to COVID-19?<br>If so can you give an example? Eg organising any new IPC activity?<br><br>Prompt: were some individual webinars more or less difficult to apply practically?	Utility and feasibility of webinar recommendations/self efficacy
Have you shared any of the information you heard in the webinars with others or do you have plans to?<br><br>Prompts: When and how was transfer of knowledge? Eg local face to face training or sharing youtube link or telegram invite? Did you share the webinar invite with anybody?	Sharing information with others
Are there other interventions/initiatives on IPC going on in your facility at the moment?<br><br>Prompt: what other IPC activities are going on as part of COVID-19 response?	Are they able to enact the learnings from the training

Supplement to: Gon G, Kessy SJ, Alimi Y, et al. Training a continent: a process evaluation of virtual training on infection prevention and control in Africa during COVID-19. *Glob Health Sci Pract*. 2023;11(2):e2200051. https://doi.org/10.9745/GHSP-D-22-00051

Is there anything else you would like webinars to cover, how and why?<br><br>Do you think the African focussed approach to the webinars adds value?<br>Prompt: is too generic, country specific better? Or does seeing other countries perspectives help you?	Utility of Africa' focus of the webinar

Supplement to: Gon G, Kessy SJ, Alimi Y, et al. Training a continent: a process evaluation of virtual training on infection prevention and control in Africa during COVID-19. *Glob Health Sci Pract*. 2023;11(2):e2200051. https://doi.org/10.9745/GHSP-D-22-00051

Supplement 2 – Focus group topic guide

Introduction:
I am ___________, an epidemiologist with ORGANISATION. As some of you may know, we are working to evaluate the Infection Prevention and Control capacity building activities run by CDC in Africa within the context of the COVID-19 pandemic.

We are conducting a short focus group discussion with you, as the implementers of the training, to gather information on how the training was delivered, whether it was delivered as intended and whether it was adapted over time. Thank you in advance, for your time.

Any information you provide during the discussion will be confidential. Your identity will not be linked to any quotes or other results of this study or included in any reports.

Can I confirm if you consent to take part in the discussion?

--

Are you happy for the discussion to be recorded? (Recordings will be kept securely, destroyed before the project ends and any data will be anonymised).

(If yes, the recording is started at this point)

Great so, to confirm, you all consent to take part in the discussion and to this discussion being recorded. Let's begin:

Topic guide:
ToT:

1. What was the aim of the training?
2. What was the content of the training?
3. How was the content developed?
4. Was it based on a needs assessment?
5. Was the content adapted over time? How?
6. Were the training sessions delivered as intended (e.g. content, format, number of trainings)?
7. What were the barriers (given the context) to implement this?

Virtual training:

8. What was the aim of the training?
9. What was the content of the training?
10. How was the content developed?
11. Was it based on a needs assessment?
12. Was the content adapted over time? How?
13. Were the training sessions delivered as intended (e.g. content, format, number of trainings)?
14. What were the barriers (given the context) to implement this?

Supplement to: Gon G, Kessy SJ, Alimi Y, et al. Training a continent: a process evaluation of virtual training on infection prevention and control in Africa during COVID-19. *Glob Health Sci Pract*. 2023;11(2):e2200051. https://doi.org/10.9745/GHSP-D-22-00051

Supplement 3 – Consent

Before we proceed, could you please read the consent statement below and let me know if you agree with the statement so we can proceed with the interview?

If you agree, please send us back an email saying that you agree with the consent statement below.

Best Regards,

IPC Evaluation Team

Interview participation email consent form

I agree to volunteer to participate in the evaluation of the Infection Prevention and Control capacity building activities run by CDC in Africa within the context of the COVID-19 pandemic, conducted by the London School of Hygiene and Tropical Medicine.
I understand this evaluation will contribute to gather feedback around how to improve the IPC webinar sessions delivered by the Africa CDC.

By returning this email, I consent the below points:

My participation to this project is voluntary. I understand that I will not be paid for my participation. I may withdraw and end the interview at anytime.

1. I agree that any information I provide during the interview will be confidential. My identity will not be linked to any quotes or other results of this study or included in any reports. All results will be presented in aggregate form, without reference to particular contexts.

2. I agree for the interview to be recorded. The recording of this interview will be kept securely, and destroyed before the project ends. Any data will be anonymised.

3. I agree for anonymous quotes from the interview to be used in publications and presentations. I agree for the data to be shared among the research partners on this project in an anonymised format.

I have read and understand the information provided to me and I voluntarily consent to participate in this evaluation.

Supplement to: Gon G, Kessy SJ, Alimi Y, et al. Training a continent: a process evaluation of virtual training on infection prevention and control in Africa during COVID-19. *Glob Health Sci Pract*. 2023;11(2):e2200051. https://doi.org/10.9745/GHSP-D-22-00051

Supplement 4 – Survey responses

Proportion who responded (out of those who attended the webinar) to the question and median of responses to the statements a) "IPC topic was clear"; b) "Slides were clear and understandable"; and c) "The facilitators explained their topics very well". Responses were out of 5; 5 indicated strongly agree and 1 indicated strongly disagree.
R= Round; S=Session; E=English; F=French; Q=Quartile

a)

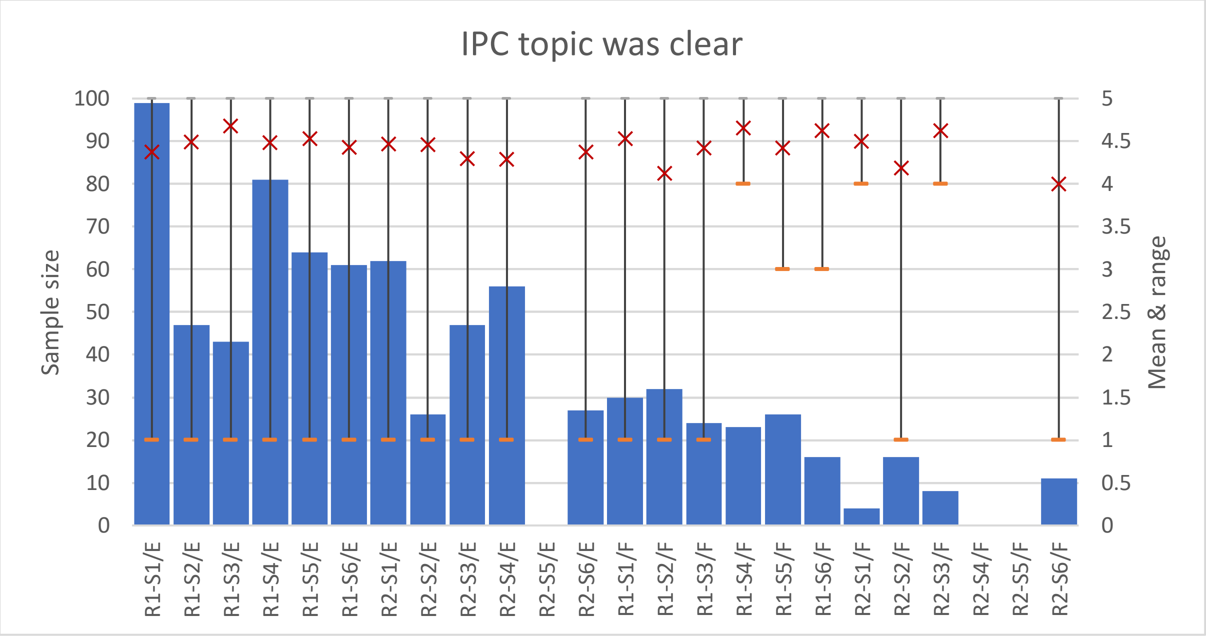

b)

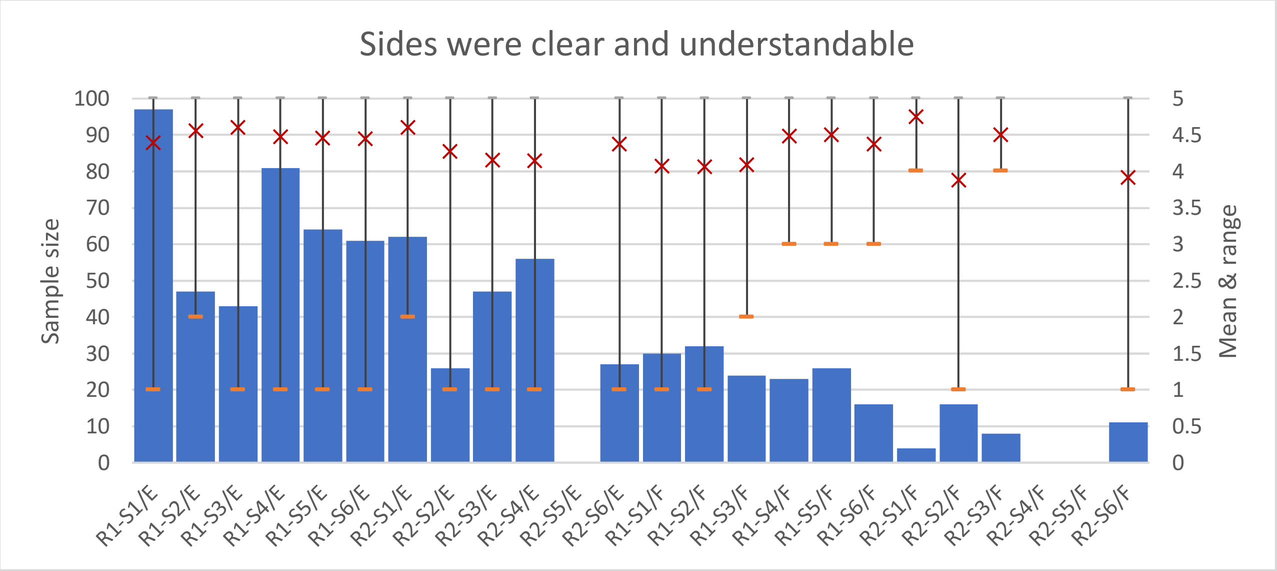

Supplement to: Gon G, Kessy SJ, Alimi Y, et al. Training a continent: a process evaluation of virtual training on infection prevention and control in Africa during COVID-19. *Glob Health Sci Pract*. 2023;11(2):e2200051. https://doi.org/10.9745/GHSP-D-22-00051

c)

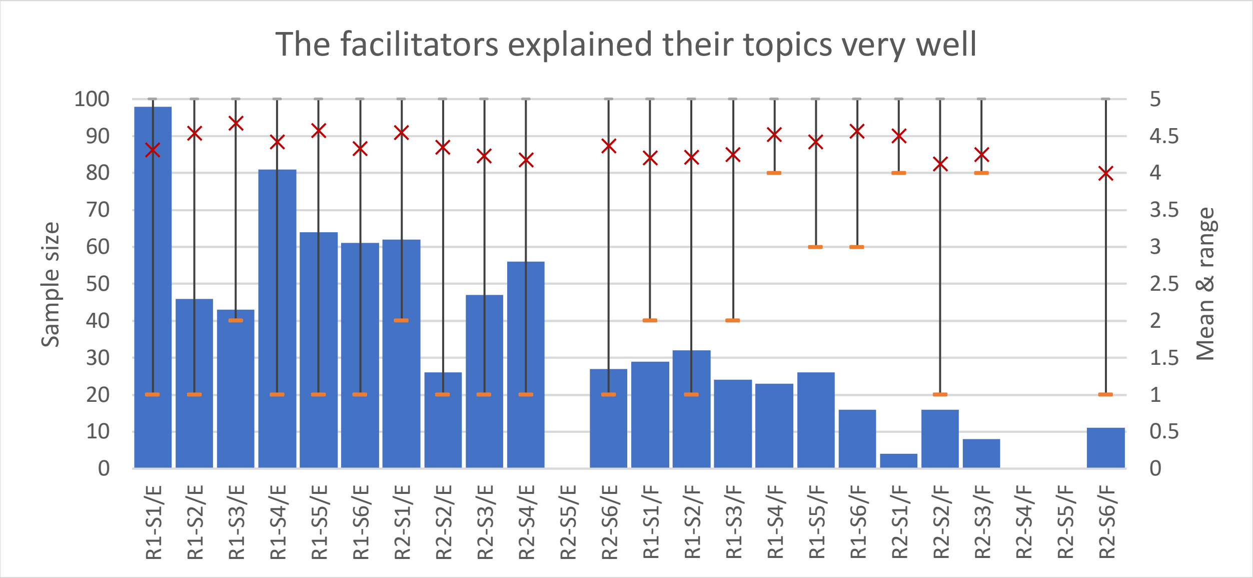

Supplement: GHSP-D-22-00051-supplements.pdf [file GHSP-D-22-00051-supplements.pdf]
